# Utility of Intraoperative Intrinsic Near-Infrared Imaging for Primary Hyperparathyroidism in Multiple Endocrine Neoplasia Type 1

**DOI:** 10.7759/cureus.55706

**Published:** 2024-03-07

**Authors:** Taisei Yasuda, Kiyomi Kuba, Eijiro Yoneyama, Masami Osaki

**Affiliations:** 1 Otolaryngology - Head and Neck Surgery, Ageo Central General Hospital, Ageo, JPN

**Keywords:** spy-elite®️ fluorescence imaging, parathyroidectomy, hyperparathyroidism, near-infrared fluorescence imaging, multiple endocrine neoplasia type1

## Abstract

Multiple endocrine neoplasia type 1 (MEN1) is an autosomal dominant disorder caused by mutations in the tumor suppressor gene *MEN1* and is characterized by parathyroid, pancreatic islet, and anterior pituitary tumors. Primary hyperparathyroidism is the most characteristic finding in MEN1, and intraoperative identification and accurate removal of the diseased parathyroid glands are vital since incomplete excision results in recurrence. This case report describes a 59-year-old woman who had pancreatic islet cell tumors and pituitary tumors and underwent selective transsphenoidal adenomectomy. Based on her medical history and examination, the diagnosis of primary hyperparathyroidism in MEN1 was made, and she underwent total parathyroidectomy with autotransplantation with SPY-Elite®️ Fluorescence Imaging (Stryker Corp., Kalamazoo, MI). Intraoperative identification of the parathyroid glands using autofluorescence with real-time intrinsic near-infrared (NIR) imaging made it easier to detect all of the parathyroid hyperplasia. After the surgery, she had hypoparathyroidism and continued with her oral calcium and vitamin D supplementation to maintain normal calcium levels during follow-up. Herein, we would like to advocate that the use of parathyroid gland autofluorescence with real-time intrinsic NIR imaging may be useful for identifying parathyroid tumors in patients with primary hyperparathyroidism in MEN1.

## Introduction

One of the treatments for multiple endocrine neoplasia type 1 (MEN1) is surgery, such as total parathyroidectomy. Intraoperative identification and accurate removal of the diseased parathyroid glands are vital since incomplete excision results in recurrence. Although several reports have shown that intraoperative parathyroid hormone (PTH) monitoring or pathological diagnosis with frozen specimens could assist in the intraoperative identification of the parathyroid glands and improve clinical outcomes, it is largely dependent on the surgeon’s experience in identifying and removing the diseased parathyroid glands [1-3]. Therefore, a simple and noninvasive device is required during surgery. The parathyroid glands produce near-infrared (NIR) autofluorescence, which enables their intraoperative identification. This technique does not require an intravenous injection of indocyanine green (ICG). Real-time intrinsic NIR imaging is useful as an intraoperative adjunctive tool and may also have significant potential for total parathyroidectomy in patients with MEN1. We used the SPY-Elite®️ Fluorescence Imaging (Stryker Corp., Kalamazoo, MI,) to detect parathyroid disease. Herein, we demonstrated its utility in intraoperative intrinsic NIR imaging in a patient with MEN1.

## Case presentation

A 59-year-old woman presented to her physician with progressive epigastric discomfort with no palpable masses. After close examination, a duodenal ulcer and hyperparathyroidism were discovered. She was referred to our hospital for consultation regarding her hyperparathyroidism. Her medical history was significant for pancreatic islet cell tumors (neuroendocrine tumor [NET]) and pituitary tumors. Thirty-two years ago, when she was 27 years old, she had undergone selective transsphenoidal adenomectomy for pituitary tumors. Subsequently, she underwent regular medical checkups for pituitary tumors and pancreatic NET, with no noted recurrence. The patient had no recent illness otherwise and was only taking a proton pump inhibitor. She had no history of allergies or smoking. Her family history included pituitary and parathyroid tumors in her father, and the patient had no siblings or children. Physical examination revealed no cervical masses. The abdomen was soft, with no organomegaly. The neurological examination findings were unremarkable. The results of her biochemistry tests were as follows: creatinine, 1.23 mg/dL; urea nitrogen, 22.7 mg/dL; calcium, 13.2 mg/dL (reference: 8.4-10.2 mg/dL); intact PTH, 1,017 pg/mL (reference: 10-60 pg/mL); thyroid-stimulating hormone, 3.20 μU/mL; free triiodothyronine, 2.54 pg/mL; and free tetraiodothyronine, 0.87 ng/dL (Table 1).

**Table 1:** Laboratory findings on admission. BUN, blood urea nitrogen; Cr, creatinine; TSH, thyroid-stimulating hormone; free T3, free triiodothyronine; free T4, free tetraiodothyronine; PTH, parathyroid hormone

Blood chemistry		Pituitary hormone		Thyroid hormone		PTH	
BUN	22.7 mg/dL	TSH	3.20 μU/mL	Free T3	2.54 pg/mL	Intact PTH	1,017 pg/mL
Cre	1.23 mg/dL			Free T4	0.87 ng/dL		
Calcium	13.2 mg/dL						

**Figure 1:**
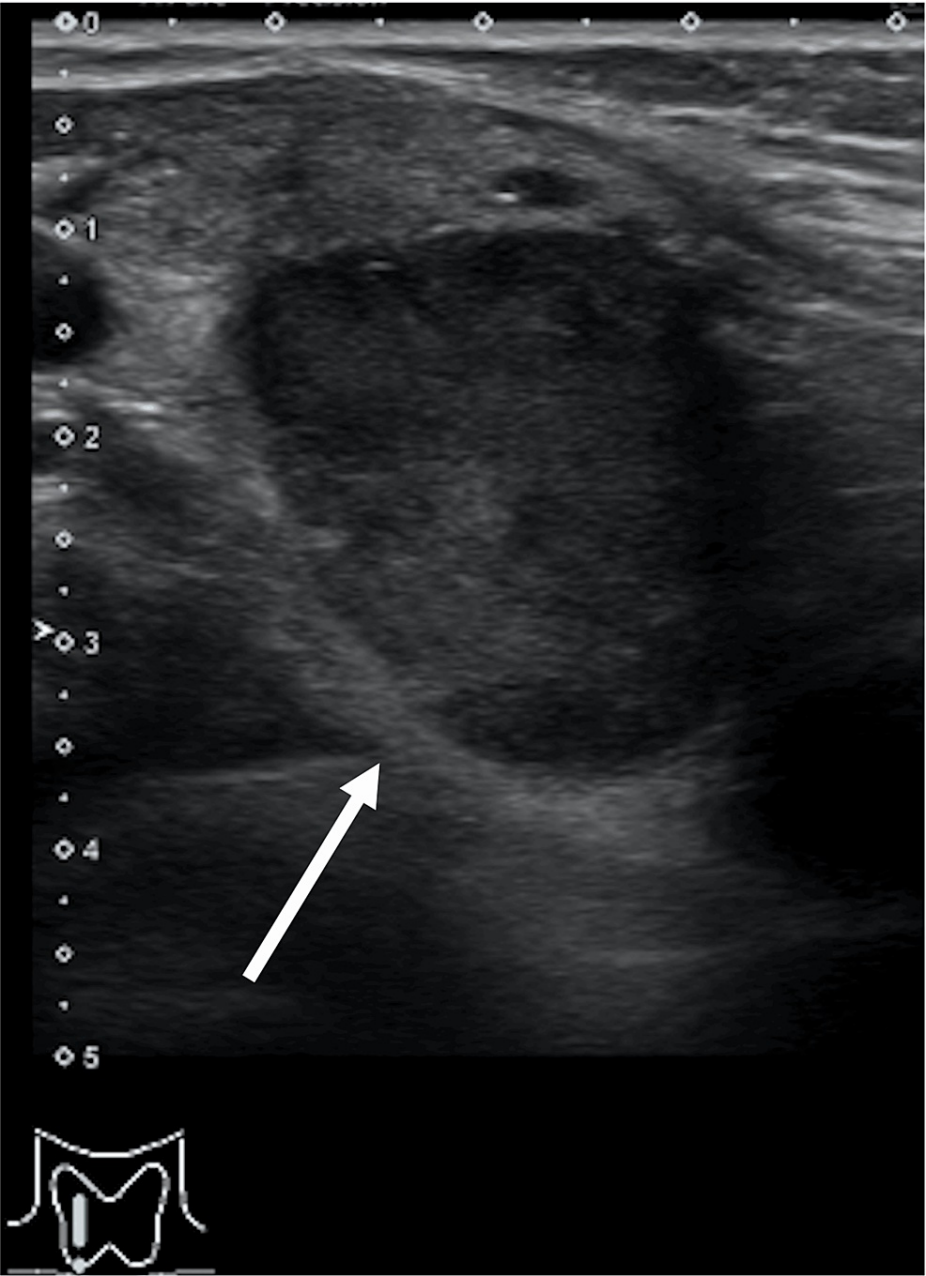
Neck ultrasonography reveals an enlarged parathyroid gland behind the lower pole of the right thyroid lobe. Approximately 2 cm in diameter (white arrow). Other parathyroid glands are not found obviously.

Her complete blood count and liver biochemistry findings were within normal ranges. Ultrasonography of the neck showed one mass approximately 2 cm in diameter in the right thyroid lobe (Figure 1).

**Figure 2:**
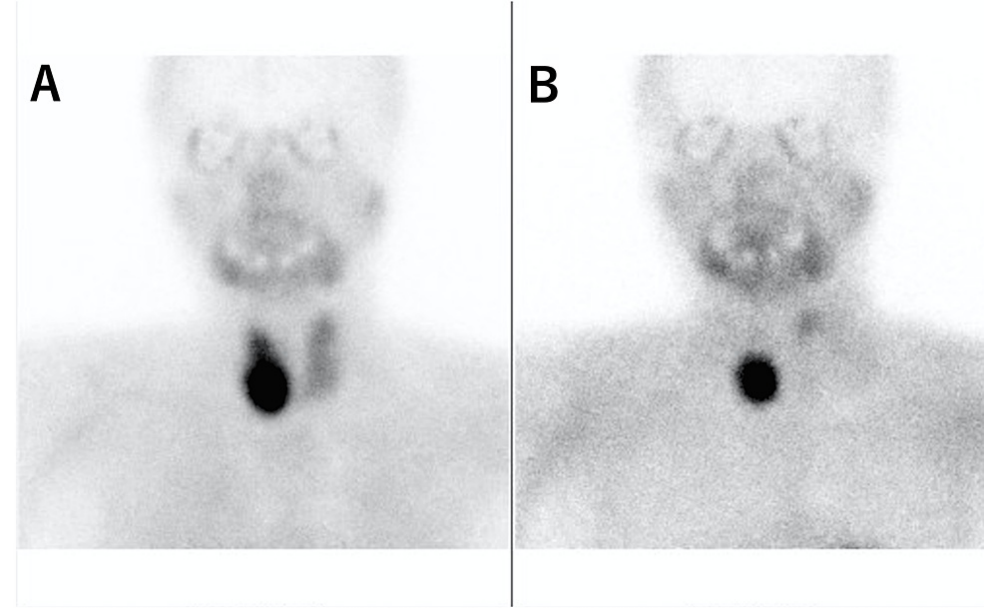
Tc-99m MIBI reveals the uptake in the right inferior parathyroid tumor and left superior parathyroid tumor. (A) Early image; (B) late image. Tc-99m MIBI, Technetium-99m methoxy-isobutyl iso-nitrile

Early and late Technetium-99m methoxy-isobutyl iso-nitrile (Tc-99m MIBI) images of her neck taken anteriorly at 20 minutes and 2 hours showed increased focal uptake, suggesting a right inferior parathyroid tumor and left superior parathyroid tumor (Figure 2).

**Figure 3:**
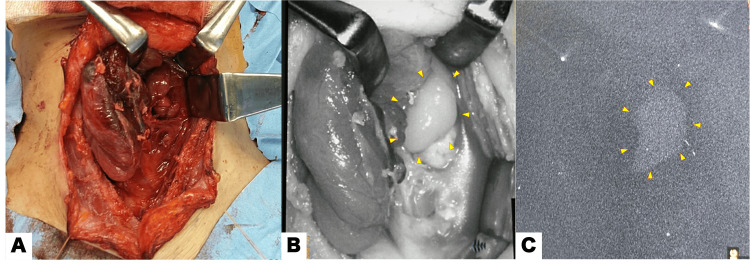
Autofluorescence in the left superior parathyroid gland of a MEN1 patient. (A) Visible light image. (B) The left superior parathyroid gland appears whiter in color than the left thyroid lobe with the SPY-Elite®️ Fluorescence Imaging (yellow arrowheads; Stryker Corp., Kalamazoo, MI,). (C) NIR light image of the left superior parathyroid gland emitting its autofluorescence (yellow arrowheads). MEN1, multiple endocrine neoplasia type 1; NIR, near infrared

According to her medical history and findings on examination, a diagnosis of primary hyperparathyroidism in MEN1 was made. We considered a genetic panel, but the patient did not want to perform the testing. We planned a total parathyroidectomy with autotransplantation with SPY-Elite®️ Fluorescence Imaging. During the procedure, a right inferior parathyroid tumor was difficult to dissect due to its high adherence to the right lobe of the thyroid gland; therefore, a right thyroid lobectomy was performed, including the removal of both superior and inferior parathyroid tumors. Next, we used SPY-Elite®️ Fluorescence Imaging to detect a left superior parathyroid tumor. The tumor was identified as white-colored autofluorescence on SPY-Elite®️ Fluorescence Imaging and was removed (Figure 3).

All of these parathyroid tumors were confirmed as parathyroid tissue by intraoperative frozen section examination. The remaining normal-sized left inferior parathyroid gland was also resected with identification by SPY-Elite®️ Fluorescence Imaging, and half of it was confirmed as a normal parathyroid gland by intraoperative frozen section, and the other half was autotransplanted into the left sternocleidomastoid muscle. The histopathological analysis of the resected specimens was consistent with that of parathyroid tumors (right superior parathyroid gland, 5 mm × 4 mm × 16 mm; right inferior parathyroid gland, 21 mm × 17 mm × 36 mm; and left superior parathyroid gland, 23 mm × 12 mm × 8 mm). On the first postoperative day, the serum total calcium and intact PTH levels dropped to 11.7 mg/dL and 24 pg/mL, respectively. However, on day 12 postoperatively, the calcium levels declined to 6.4 mg/dL although high doses of oral and intravenous calcium along with calcitriol supplementation. Intact PTH was not detected. We then considered this as a case of hungry bone syndrome. She continued with her oral calcium and vitamin D supplementation to maintain normal calcium levels during follow-up.

## Discussion

MEN1 is an autosomal dominant disorder caused by mutations in the tumor suppressor gene *MEN1* and is characterized by parathyroid, pancreatic islet, and anterior pituitary tumors [4]. Primary hyperparathyroidism is the most characteristic finding in MEN1, occurring in approximately 95% of all cases [5].

In primary hyperparathyroidism, vague symptoms associated with hypercalcemia (e.g., polyuria, polydipsia, constipation, and malaise) may occur. By increasing the circulating PTH concentrations, calcium is mobilized through bone resorption, and calcium resorption from the distal renal tubule is enhanced by 1,25-dihydroxycholecalciferol. PTH also induces 1α-hydroxylase (CYP27B1) in the proximal renal tubules, which enhances 1,25-dihydroxycholecalciferol production. PTH promotes the renal excretion of phosphate and bicarbonate ions generated during bone resorption, resulting in an almost exclusive calcium mobilization into the cellular fluid. Excess PTH causes hypercalcemia, hypokalemia, and metabolic acidosis. In this case, the serum intact PTH level was 1,017 pg/dL on our first consultation, and the patient had asymptomatic hypercalcemia (13.2 mg/dL).

Subtotal or total parathyroidectomy is the definitive treatment in patients with MEN1; however, it is unclear which surgery is better. Since all four parathyroid glands are usually affected by multiple adenomas or hyperplasia, parathyroid tumors should be carefully removed. In our case, we performed a total parathyroidectomy with autotransplantation. This technique aims to radically remove all parathyroid glands, including the occult glands, and avoid cervical recurrences that are difficult to treat due to scarring. In general, autologous transplantation of normal parathyroid glands can reduce the complications of hypocalcemia caused by low PTH. This technique has been reported to reduce the risk of persistent hyperparathyroidism [6] and provides longer recurrence-free survival [7] compared to subtotal parathyroidectomy, but has a higher risk of hypoparathyroidism [6].

Intraoperative identification of the parathyroid glands using autofluorescence with real-time intrinsic NIR imaging appears to be useful in patients with primary hyperparathyroidism [8]. Several reports noted the efficacy of intraoperative PTH monitoring or intraoperative pathological diagnosis with frozen specimens [1,2], but NIR (such as in this case, through the use of the SPY-Elite®️ Fluorescence Imaging) may make it easier to detect all of the parathyroid hyperplasia without invasion, aiding in its complete removal, and producing a lower risk of recurrence. Real-time intrinsic NIR imaging does not require an intraoperative blood test for PTH measurements or intravenous injection of ICG. SPY-Elite®️ Fluorescence Imaging is a NIR video camera system used for laser angiography using ICG designed to detect tissue perfusion; the ICG dye binds to plasma proteins and emits fluorescence when excited by an 805-nm laser [9]. On the other hand, parathyroid autofluorescence imaging shows that the parathyroid gland produces an autofluorescence at 820-830 nm with a 785 nm excitation light [10]. The similarity between parathyroid autofluorescence and the fluorescent property of ICG is the reason why SPY-Elite®️ Fluorescence Imaging allows for the visualization of parathyroid fluorescence. In particular, parathyroid fluorescence is 2 to 11 times higher than that of the thyroid tissues, with peak fluorescence occurring at 820-830 nm [10]. It should be noted that as brown fat can fluoresce and decrease parathyroid autofluorescence in patients with MEN1, the rates of false-positive and false-negative autofluorescence may be higher in such patients [8]. Therefore, real-time intrinsic NIR imaging is only an auxiliary tool. We first detected the parathyroid tumors through the naked eye with reference to the preoperative Tc-99m MIBI, ultrasonography, and neck computed tomography and then used SPY-Elite®️ Fluorescence Imaging as an intraoperative confirmation.

There are potential limitations for using NIR imaging such as SPY-Elite®️ Fluorescence Imaging. First, a device such as SPY-Elite®️ Fluorescence Imaging costs a lot, so all facilities do not have one. However, the facility where surgeons conduct gastrointestinal, cardiothoracic, and reconstructive surgery on the breast and other body parts may have NIR imaging accessible at no charge. Second, the specificity and sensitivity of SPY-Elite®️ Fluorescence Imaging are not known. Kahramangil et al. observed that the sensitivity of NIR imaging for detecting parathyroid gland autofluorescence was high and ranged between 97% and 99% [11]. In the report, the resulting fluorescence was captured using the Fluobeam device (Fluoptics, Grenoble, France), not SPY-Elite®️ Fluorescence Imaging. To our knowledge, this is the first time using SPY-Elite®️ Fluorescence Imaging to detect autofluorescence. Our report lacks a statistical analysis and only describes the usefulness of SPY-Elite®️ Fluorescence Imaging in a single MEN1 case at a single institution. Since there are limitations to stating usefulness, we need to accumulate more cases in the future.

## Conclusions

Using parathyroid gland autofluorescence with real-time intrinsic NIR imaging is useful for identifying parathyroid tumors in patients with primary hyperparathyroidism. Specifically, we highlight that real-time intrinsic NIR imaging could be a valuable noninvasive tool not only in parathyroidectomy for primary and secondary hyperparathyroidism in patients diagnosed with non-MEN1 but also in subtotal and total parathyroidectomy in MEN1 patients, where all thyroid hyperplasia should be removed without excess or deficiency.
